# CircRUNX2 through has‐miR‐203 regulates RUNX2 to prevent osteoporosis

**DOI:** 10.1111/jcmm.13888

**Published:** 2018-10-16

**Authors:** Qudong Yin, Jian Wang, Qiang Fu, Sanjun Gu, Yongjuan Rui

**Affiliations:** ^1^ Department of Orthopaedics Wuxi No. 9 People's Hospital Affiliated to Soochow University Wuxi Jiangsu China; ^2^ Department of Laboratory Jiangsu Institute of Parasitic Disease Wuxi Jiangsu China

**Keywords:** circRUNX2, MiR‐203, osteoporosis, RUNX2

## Abstract

**Objective:**

We aimed to discover the molecular mechanism of hsa_circ_0076694 (circRUNX2) on osteogenic differentiation. We also explored the interaction between circRUNX2, miR‐203 and RUNX2.

**Methods:**

Clinical samples obtained from femoral neck fracture patients’ bone tissues were used to collect circRUNX2, miR‐203, and RUNX2 expression data, while their expression changes were observed in human bone mesenchymal stem cells (hBMSCs) during osteogenic differentiation. QRT‐PCR and Western blot were used to analyse levels of RNAs and proteins. Biotin pull down, RIP, RNA FISH, and Dual‐Luciferase Reporter assays demonstrated the relationship between circRUNX2, miR‐203, and RUNX2. ALP and ARS staining were used to measure the degree of osteogenic differentiation under the control of circRUNX2, miR‐203.

**Results:**

CircRUNX2 were down‐regulated in osteoporotic patients’ bone tissues. CircRUNX2 could inhibit miR‐203 expression by sponging miR‐203. MiR‐203 inhibited osteogenic differentiation by targeting the 3′‐UTR of RUNX2 and down‐regulate RUNX2 expression. Overexpression of circRUNX2 promoted the expression of osteogenic differentiation‐related proteins such as RUNX2, OCN, OPN, BSP, and prevented osteoporosis.

**Conclusion:**

circRUNX2 could sponge miR‐203 and enhance RUNX2 expression, thus circRUNX2 prevents osteoporosis and may provide a novel therapeutic strategy for it.

## 1 INTRODUCTION

Osteoporosis is defined by the World Health Organization as a bone mineral density of about 2.5 standard deviations below the mean bone mass (average of young, healthy adults) as measured by dual‐energy x‐ray absorptiometry.^1^ It is a skeletal disease characterized by low bone mass, defective bone structure, and high risks of fracture even under minor pressure.^2^ In the world, about 200 million people suffered from the osteoporosis, and the insufferable pain and possible complications of which affect the life quality of patients greatly.^3^ Ingestion of calcium, vitamin D, bisphosphonates are often adopted to prevent and cure osteoporosis.^1^ However, to cure it thoroughly, we are focusing on how to find a microscopic therapy for osteoporosis which is from the interatcion among the genes, besides the common physical and medical treatment.

Recently much attention has been paid to Circular RNAs (circRNAs), which is one of a class of noncoding RNAs, with circular structure by joining the 3′end of the RNA to the 5′end.^4^ They were important in regulating the expression of other genes. For example, Liu et al found 2:27713879|27755789 and 2:240822115|240867796 participated in regulation network in osteogenic differentiation.^5^ Also, they were able to affect the expression of some essential carcinogenic mRNAs by targeting miRNAs and changing the miRNAs expression which would subsequently change the miRNAs level.^6^

It was predicted that circRNAs would combine with miRNA and compete for the binding sites on the miRNA with mRNAs.^7^ CircRNAs expression would alter the mRNA expression by targeting miRNAs that were binding to the mRNAs chosen. For instance, it is reported that hsa_circRNA_103801, a novel circRNA, targeted genes which were mainly involved in biological processes and cellular components.^8^ CircUBAP2 inhibited the expression of microRNA‐143 (miR‐143) giving rise to promote osteosarcoma growth and inhibit apoptosis both in vitro and in vivo.^9^ Also, circRNA_0009910 was found to be overexpressed in osteosarcoma cells (OS cells), and knocking down of which inhibited cell proliferation, and induced cell cycle arrest as well as apoptosis in OS cells. It worked by targeting miR‐449a, which had the opposite effect on OS cells.^10^ These researches provide us great examples to deem that miRNA‐circRNA interaction is considered to one of better direction for therapy.

Runt related transcription factor, known as RUNX factors, which proteins located in sub‐nuclear domains and integrated cell signals during formation of gene promoter regulatory complexes.^11^ RUNX2 is a member of the human RUNT related transcription factor family essential during embryogenesis for skeletal development. It was recognized on account of its oncogenic properties and many studies showed that a de‐regulating of RUNX2 function leads to progression and invasion of different tumours.^12^ Also, RUNX2 was reported to closely relate to bone formation and hypertrophic chondrocyte differentiation.^13^ Some evidence showed that the down‐regulated RUNX2 protein expression inhibited bone formation, and decreased bone mass.^14^ Although there were already some researches on RUNX2‐related regulation in osteogenic differentiation, the understanding towards to the mechanism of it was far from being comprehensive. Herein, we wanted to investigate the molecule mechanism of RUNX2 in the osteogenic differentiation.

In this study, we tried to find the expression levels of miR‐203, circRUNX2, and RUNX2 in osteoporotic patients and how miR‐203, circRUNX2, and RUNX2 affect each other expression levels to influence the development of the osteoporosis.

## 2 MATERIALS AND METHODS

### 2.1 Clinical samples

Trabecular bone samples from the trochanteric region of the femur at a site far from the periarticular bone were obtained from 20 patients undergoing hip replacement because of femoral neck fracture. A total of 10 osteoporotic specimens were collected from women aged 62‐89 (average age: 75). Ten nonosteoporotic specimens derived from women patients with suffered external traumatic fracture. To keeping samples fresh, frozen environment was needed. None of the patients underwent any medical treatment that affected bone or mineral metabolism before enrolling in this study. The study was authorized by the Ethics Committee of Wuxi No. 9 People's Hospital Affiliated to Soochow University, and all patients were offered informed consents and signed for those confirmations.

### 2.2 Cell culture and induction of osteogenic differentiation

Human bone mesenchymal stem cells (hBMSCs) were bought from the BeNa Culture Collection (BNCC, Beijing, China). HBMSCs were cultured in minimum essential medium Alpha Medium (α‐MEM) supplemented containing 10% FBS, 2Mm L‐Glutamine, 100 U/mL penicillin and 100 μg/mL streptomycin and stored in a humidified atmosphere of 5% CO_2_ at 37°C. Osteogenic (OS) differentiation of hBMSCs was induced according to a previously published protocol.^15^ The OS medium was supplemented with osteogenic inducers: including 10‐8 mol L^−1^ dexamethasone (Dex); ascorbic acid 2‐phosphate (AsAP) with a concentration of 50 μg/mL; and 10 m mol L^−1^ Glycerol 2‐phosphate (Gly) (Sigma, USA). HBMSCs were seeded at density of 5 x 10^5^ cells/well in a 12‐well plate, and the culture medium was replaced with an OS medium when the cells reached 80% confluence. Moreover, OS medium were changed every 3 days, and then induced cells were harvested and analysed at 0, 7, 14, and 21 days.

### 2.3 Cell transfection

MiR‐203 mimics, miR‐203 inhibitor, miRNA control, plasmid mediated circRUNX2 overexpression, circRUNX2 siRNA, and control vector were purchased from Genechem (Shanghai, PR, China). HBMSCs were planted in 6‐well plates for 24 hours prior to the transfection with 50%‐60% confluence, and then were transfected with Lipofectamine 2000 (Invitrogen, Carlsbad, CA, USA) according to the manufacturer's instructions.

### 2.4 QRT‐PCR

Before extract of total RNA, the bone is rapidly placed in the mortar that is precooled with liquid nitrogen, and then repeatedly ground to the powder. Total RNA was extracted using TRIzol reagent (ThermoFisher, China) and cDNA was synthesized using iScript™ cDNA Synthesis Kit (Biorad, China) following the kit's protocol. iQ™ SYBR Green supermix (Biorad, China) was used to perform QRT‐PCR. 7500 HT Fast Real‐Time PCR System (Applied Biosystems) was used to cycle and quantify reactions. Relative gene expression levels of circRUNX2, miR‐203 and *RUNX2* were evaluated. U6 and GADPH were used as miRNA and long RNA endogenous normalization controls. Primers for RT‐PCR were supplied in Table 1.

**Table 1:** Primer sequences used for qPCR

S	Primer sequences
MiR‐203	F: 5′‐ GATCGATCACCAGGATTTG‐3′
MiR‐203	R: 5′‐ GTATCCAGTGCGAATACCTC‐3′
U6	F: 5′‐ CTCGCTTCGGCAGCACA‐3′
U6	R: 5′‐ AACGCTTCACGAATTTGCGT‐3′
RUNX2	F: 5′‐ GTATCCAGTGCGAATACCTC ‐3′
RUNX2	R: 5′‐ GTATCCAGTGCGAATACCTC ‐3′
CircRUNX2	F: 5′‐ATCCACTCTACCACCCCGCT‐3′
CircRUNX2	R: 5′‐ GTCATAGGACCACGGCGG ‐3′
GAPDH	F: 5′‐ TCGGAGTCAACGGATTTGGT‐3′
GAPDH	R: 5′‐ TTCCCGTTCTCAGCCTTGAC‐3′

F: forward primer; R: reverse primer.

### 2.5 Western blotting

Bone tissue protein extraction kit (BestBio, ShangHai, China) was used to extract the protein in clinical bone tissues. Proteins in hBMSCs were extracted by total protein extraction kit (BestBio, ShangHai, China). And total protein concentration was determined with a protein assay kit (Beyotime, ShangHai, China) following the manufacturer's instructions. Total protein (10 μg) was boiled for 5 minutes in 1× loading buffer, chilled on ice, and then separated on 12% SDS‐PAGE and transferred into polyvinylidene fluoride (PVDF) membranes (Millipore, Bedford, MA, USA). Nonspecific protein interactions were blocked by incubating with 5% fat‐free milk in TBST buffer (50 m mol L^−1^ Tris‐HCl, 150 m mol L^−1^ NaCl, 0.05% Tween 20, pH 7.6) for 1 hour at 4°C. The membranes were incubated overnight with primary antibody for anti‐RUNX2 (ab23981, 1 μg/mL), anti‐ALP (ab229126, 1: 1000), anti‐OPN (ab216406, 1:1000), anti‐OCN (ab93876, 1:500), anti‐BSP (ab52128, 1:500), or anti‐GAPDH (ab9485, 1:2500) primary antibody at 4°C. Unbound antibodies were removed by washing in TBST buffer three times (10 min/wash). Then the membranes were incubated with secondary antibody horseradish peroxidase labelled goat anti‐rabbit IgG (ab6721, 1:5000) at room temperature (RT) for 1 hour, then washed with TBST buffer three times (10 min/wash). The blots were visualized using ECL according to the instructions (Millipore).

### 2.6 Biotin pull down assay

The pull‐down assay with biotinylated RNA was performed as described.^16^, ^17^ In brief, for circRUNX2 pulled down miRNAs, the biotinylated‐circRUNX2 probe was incubated with C‐1 magnetic beads (Life Technologies, Carlsbad, CA, USA) to generate probe‐coated beads, then incubated with sonicated HBMSCs at 4°C overnight, followed by eluted and qRT‐PCR. For miR‐203 pulled down circRUNX2, hBMSCs with circRUNX2 overexpression were transfected with biotinylated miR‐203 mimics or mutant using Lipofectamine 2000. The cells were harvested, lysed, sonicated, and incubated with C‐1 magnetic beads (Life Technologies, Carlsbad, CA, USA), followed by washed and qRT‐PCR.

### 2.7 RNA binding protein immunoprecipitation (RIP) assay

RNA binding protein immunoprecipitation (RIP) assay was performed using the Magna RIP Kit (Millipore, USA) and Ago2 antibody (Cell Signaling Technology, USA) in accordance with the manufacturer's instructions. In brief, 10^7^ transfected cells were washed in ice‐cold PBS twice, lysed in an equal volume of RIP lysis buffer and then incubated with 5 μg of primary antibodies for 2 hours at 4°C. Subsequently, 50 μL of prepared magnetic beads was added to each sample and incubated at 4°C overnight. The beads were washed briefly with RIP buffer for five times and resuspended in 500 μL of TRIzol LS (Life Technology, USA). The binging products were detected by qRT‐PCR.

### 2.8 RNA fluorescence in situ hybridization (FISH)

The RNA fluorescence in situ hybridization assay was performed by using Fluorescent In Situ Hybridization Kit (RiboBio, Guangzhou, China) according to the manufacturer's guidelines. And Cy3‐labelled circHIPK3 probes and Dig‐labelled locked nucleic acid miR‐203 probes (Ribo‐Bio, Guangzhou, China) were measured by the Fluorescent in Situ Hybridization Kit, followed by visualized with a confocal microscopy.

### 2.9 Dual‐Luciferase reporter assay

A mixture of 500 ng wild‐type vector (Yeasen, Shanghai, China) or mutant vector with 20 n mol L^−1^ miR‐203 mimics or miR‐NC were cotransfected into 293 (BNCC, Beijing, China) cells by applying Lipofectamine 2000 (Invitrogen) under the manufacturer's instructions. Then we harvested cells 48 hours after transfection and measured luciferase activity using the Dual Luciferase Reporter Assay System (Yeasen, Shanghai, China).

### 2.10 Alkaline phosphatase staining (ALP)

After osteogenic differentiated 7 days or 14 days, cells were washed with DPBS (Dulbecco's Phosphate Buffer Saline) and fixed for 1 hour with 10% Neutral Buffered Formalin (NBF). After washing with DPBS, cells were added 500 μL of BCIP/NBT liquid substrate and incubated in dark at room temperature for 10 minutes. Images were taken in the end. After each period, the cells were washed, lysed and incubated with buffered substrate (p‐NPP: Carbonate buffer, pH 10.3: 2 m mol L^−1^ MgCl_2_: 1:1:1) for 1 hour at 37˚C. The reaction was stopped by adding 3N NaOH and ALP activity reflected by measuring p‐nitrophenol (p‐NP) absorbance was determined at 405 nm wavelength with microplate reader.

### 2.11 Alizarin red staining

Alizarin red staining (ARS) was performed to detect the osteoblast calcification. For ARS, hBMSCs were seeded at a density of 5 × 10^4^ cells/well in 12 well plates and cultured for 24 hours for transfection before adding differentiation medium. Then cells were washed with PBS twice, fixed with 95% ethanol for 10 minutes, washed with distilled water for three times, and followingly stained by Alizarin red S staining solution (Cyagen, China) with PH 8.3 for 30 minutes at 37°C. After being rinsed twice with distilled water, the cells were photographed.

### 2.12 Statistical analysis

All experiments were repeated at least three times. Data were analysed using Graph Pad Prism 6.0 software. All data were presented as mean ± SD. Differences between two groups were identified by unpaired *t* test, and differences between more than two groups were analysed using one‐way analysis of variance (ANOVA). Moreover, Spearman correlation analysis was used to detect the relationship between molecules. *P *<* *0.05 was taken as statistically significant.

## 3 RESULTS

### 3.1 CircRUNX2 and RUNX2 was lowly expressed in osteoporotic patients

**Figure 1:**
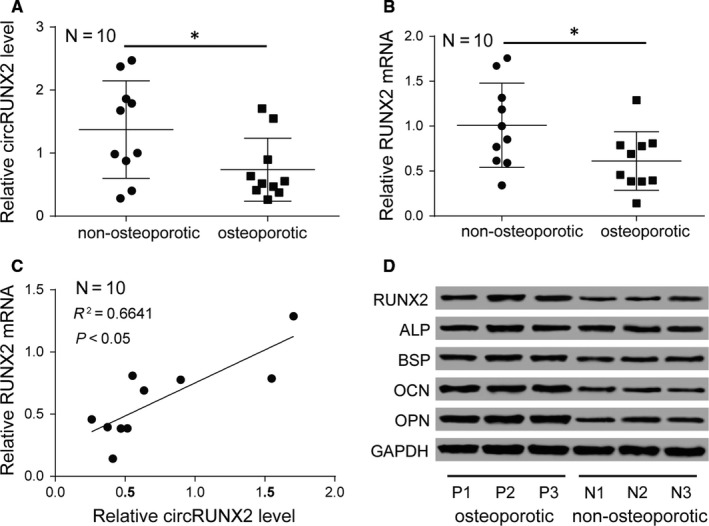
CircRUNX2 and RUNX2 were both low expression in osteoporotic tissues. (A and B) Expression of circRUNX2 and RUNX2 were lower in osteoporotic tissues. N represents clinical sample number. *P <* *0.05. C, Spearman correlation analysis showed a positive correlation relationship in the expression of circRUNX2 and RUNX2 with 10 tissues and *r* ^2^ of 0.6641. D, Western blot detected RUNX2, ALP, BSP, OCN, OPN expressions in randomly selected three osteoporotic tissues and three nonosteoporotic tissues

CircRUNX2 and RUNX2 mRNA expression levels was found to be lower in osteoporotic patients’ bone tissues than nonosteoporotic bone tissues (Figure 1A,B). Spearman correlation analysis analysed correlation between circRUNX2 and RUNX2 which showed a positive relationship (Figure 1C). In addition, proteins of RUNX2, ALP, BSP, OCN, and OPN were detected by western blotting in osteoporotic and nonosteoporotic bone tissues. Results showed protein levels of RUNX2, BSP, OCN, and OPN was lower in osteoporotic bone tissues than that in nonosteoporotic bone tissues and ALP protein level was no obvious difference between osteoporotic and nonosteoporotic bone tissues (Figure 1D).

### 3.2 MiR‐203 contained the targets of circRUNX2 and RUNX2

**Figure 2:**
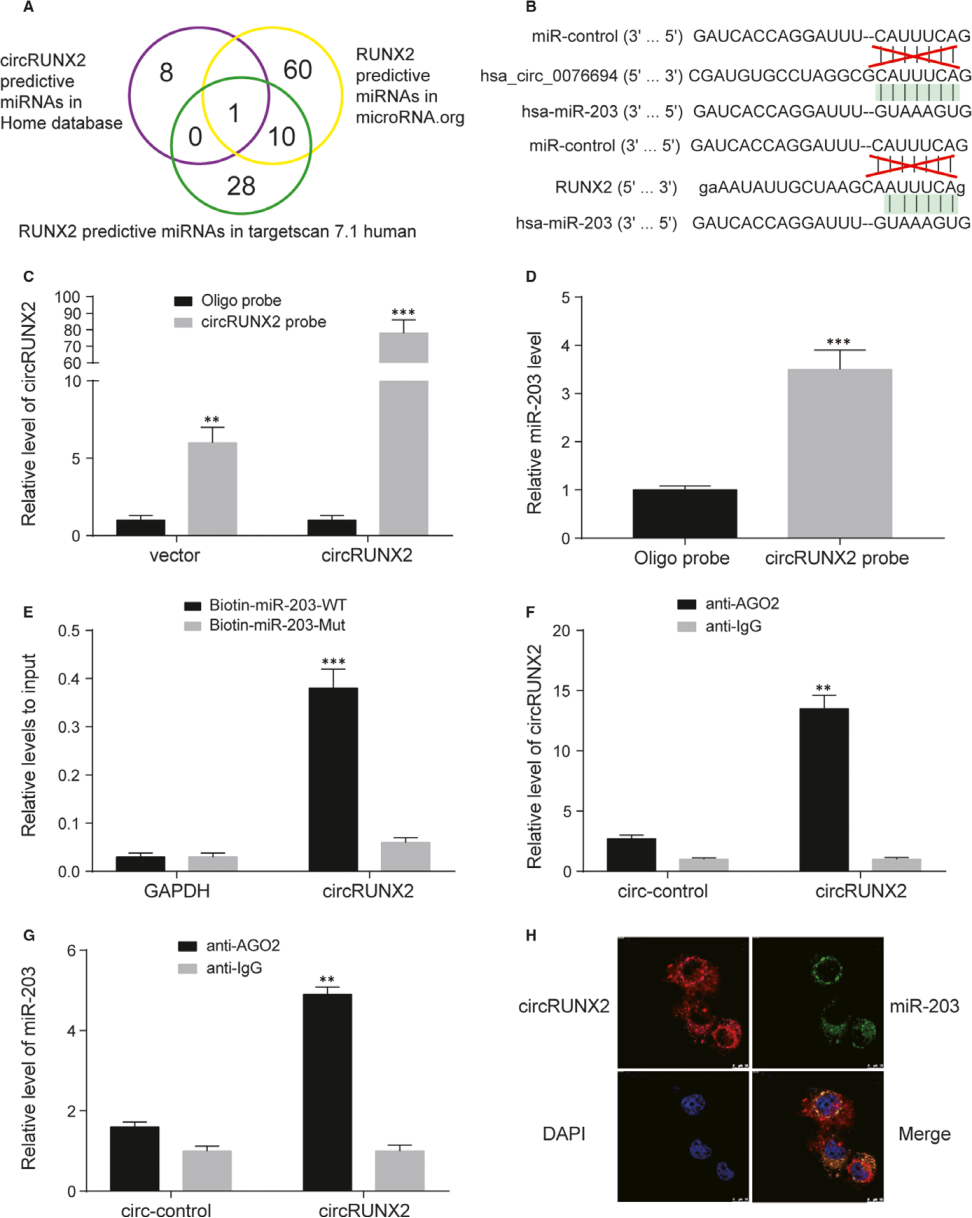
MiR‐203 targeted the 3′UTR of circRUNX2 and RUNX2. A, The Venn diagram showed the predictive miRNAs targeted circRUNX2 (Circinteractome) and RUNX2 (predicted in microRNA.org and TargetScan 7.1 human). B, The detailed sequence information of miR‐203 complemented with has_circ_0076694 (circRUNX2) and RUNX2. C, Lysates from hBMSCs with circRUNX2 overexpression were subject to biotinylated‐circRUNX2 pull down assay and the expression levels of circRUNX2 were detected by qRT‐PCR. ***P <* *0.001, **P <* *0.01. D, The expression of miR‐203 were quantified by qRT‐PCR after the biotinylated‐circRUNX2 pull down assay in hBMSCs. ***P <* *0.001. E, The biotinylated wild‐type/mutant miR‐203 was, respectively, transfected into hBMSCs with circRUNX2 overexpression. ***P <* *0.001. (F‐G) AGO2 RNA immunoprecipitation (RIP) assay for the levels of circRUNX2 and miR‐203 in hBMSCs overexpressing circRUNX2 or circ‐control. **P <* *0.01. H, Fluorescence in situ hybridization assay was conducted to determine the co‐location between circRUNX2 and miR‐203. Scare bar = 20 μm

MiRNAs targeting circRUNX2 were predicted by Home database. Moreover, miRNAs targeting RUNX2 were predicted in microRNA.org and in Target Scan 7.1 human. Finally, miR‐203 was the only one predictive miRNAs in the intersection of three kind of predictive miRNAs (Figure 2A). Hsa‐circ‐0076694 RNA and RUNX2 mRNA derived from the same parent gene, RUNX2 gene, contained the same binding seed region (UGAAAU) of miR‐203 (Figure 2B). A 3′ terminal‐biotinylated‐circRUNX2 probe was designed to the interact between circRUNX2 and miR‐203. The probe was verified by the enrichment of circRUNX2 in hBMSCs and circRUNX2 overexpression increased the enrichment efficiency (Figure 2C). qRT‐PCR result showed miR‐203 was obviously pull downed by circRUNX2 probe in hBMSCs (Figure 2D). To further confirm the direct binding of miR‐203 and circRUNX2, biotin‐labelled miR‐203 and its mutant mimics were used to pull down circRUNX2 in hBMSCs, the results revealed that wild‐type miR‐203 captured more circRUNX2 compared with the mutant (Figure 2E). For further study, AGO2 RNA immunoprecipitation assay was performed. The result of qRT‐PCR showed more circRUNX2 and miR‐203 were pulled down by AGO2 compared with IgG in hBMSCs (Figure 2F,G). Moreover, RNA fluorescence in situ hybridization (FISH) assay was applied to assess whether there is a colocation between circRUNX2 and miR‐203, and the result suggested circRUNX2 and miR‐203 were colocated in cytoplasm (Figure 2H).

**Figure 3:**
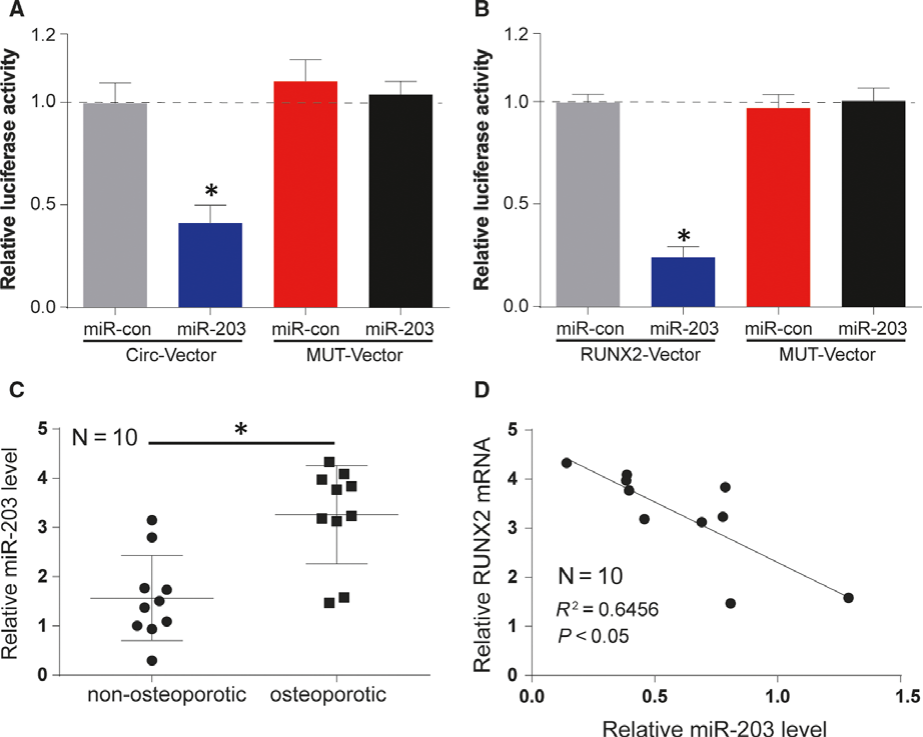
MiR‐203 targeted the 3′UTR of circRUNX2 and RUNX2. (A and B) Dual luciferase report detected the link between circRUNX2/RUNX2 and miR‐203. *P <* *0.05. (C and D) The level of miR‐203 was higher in osteoporotic tissues and had a negative correlation relationship with RUNX2 in 10 osteoporotic tissues (*r* ^2^ = 0.6456). *P <* *0.05

Following, dual‐Luciferase Reporter assay was performed to verify the target relationships of miR‐203 and circRUNX2/RUNX2 (Figure 3A,B). The results implied the target relationships with the phenomenon that miR‐203 reduced the luciferase activity in the group of wild‐type circular RNA (WT‐circ) and wild‐type RUNX2 3′UTR (WT‐RUNX2). MiR‐203 level was also detected in osteoporotic patients, and osteoporotic patients were with a higher miR‐203 level compared to nonosteoporotic patients (Figure 3C). Furthermore, the negative correlation relationship between RUNX2 mRNA and miR‐203 were observed by Spearman correlation analysis in 10 clinical samples (Figure 3D).

### 3.3 CircRUNX2 and miR‐203 affected osteogenic differentiation through regulating RUNX2

**Figure 4:**
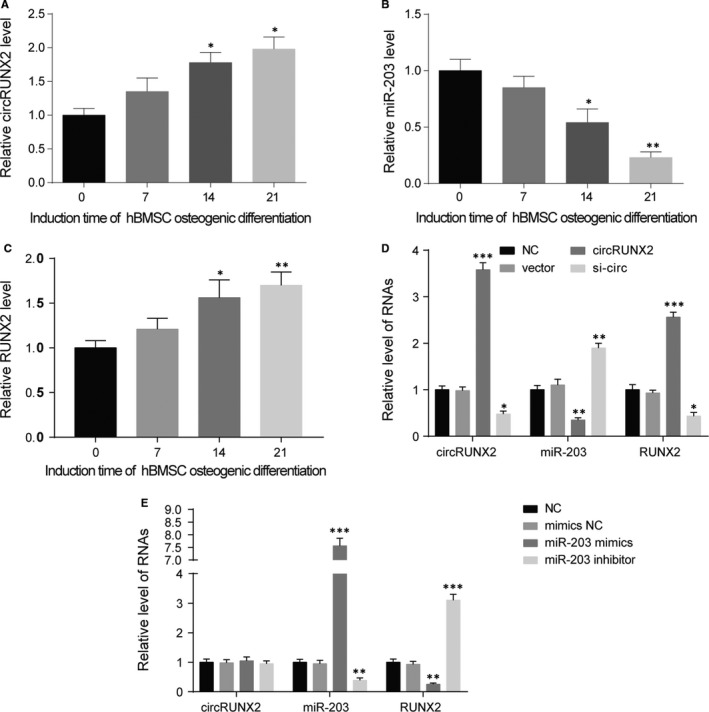
CircRUNX2 affected osteogenic differentiation through regulating miR‐203. (A, B and C) CircRUNX2 and RUNX2 expressions were up‐regulated, but miR‐203 expression was down‐regulated through inducing hBMSC osteogenic differentiation. **P <* *0.01, *P <* *0.05. D, Overexpressed circRUNX2 increased RUNX2 expression level and decreased miR‐203 expression level in hBMSCs. ***P <* *0.001, **P <* *0.01, *P <* *0.05. E, RUNX2 expression was inhibited by up‐regulation of the miR‐203 and promoted by down‐regulation of the miR‐203 in hBMSC. ***P <* *0.001, **P <* *0.01

The expression levels of circRUNX2, miR‐203, and RUNX2 were detected at the 0 day, 7th days, 14th days, and 21th days of osteogenic differentiation. During hBMSCs osteogenic differentiation, circRUNX2 and RUNX2 expressions showed an increasing trend, but miR‐203 expression was gradually decreased (Figure 4A,B,C). To further investigate the regulation links between circRUNX2, miR‐203, and RUNX2, qRT‐PCR assays were performed after regulating circRNUX2, miR‐203, or RUNX2. Results showed that, in hBMSCs, circRUNX2 overexpression lead to the reduction in miR‐203 and the increase in RUNX2 mRNA, circRUNX2 down‐regulation caused the opposite results (Figure 4D). MiR‐203 up‐regulation inhibited RUNX2 expression, and miR‐203 down‐regulation promoted RUNX2 expression. In Addition, circRUNX2 was not regulated by miR‐203 (Figure 4E). Similarly, circRUNX2 and miR‐203 were not regulated by RUNX2 (Figure S1A).

**Figure 5:**
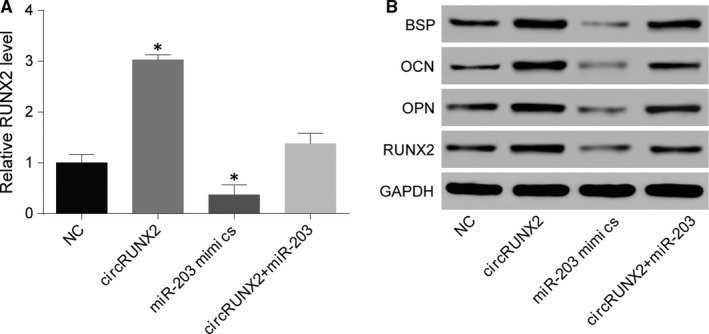
CircRUNX2 affected osteogenic differentiation through regulating miR‐203. (A and B) RUNX2 mRNA level and bone differentiation related proteins, RUNX2, BSP, OCN and OPN, were detected by qRT‐PCR and WB. *P <* *0.05

Additionally, hBMSCs with four kinds of various treatments, namely NC group, circRUNX2 overexpression group, miR‐203 overexpression, and co‐overexpression of circRUNX2 and miR‐203, were applied to survey the effects of circRUNX2, miR‐203, and RUNX2 on osteogenic differentiation. The result of qRT‐PCR confirmed again miR‐203 overexpression reduced RUNX2 expression and RUNX2 expression was no obvious change after co‐overexpression of circRUNX2 and miR‐203 (Figure 5A). Moreover, RUNX2 and other bone differentiation related proteins, BSP, OCN, and OPN determined by Western blot, were increased by circRUNX2 overexpression, but were decreased by miR‐203 up‐regulation (Figure 5B).

**Figure 6:**
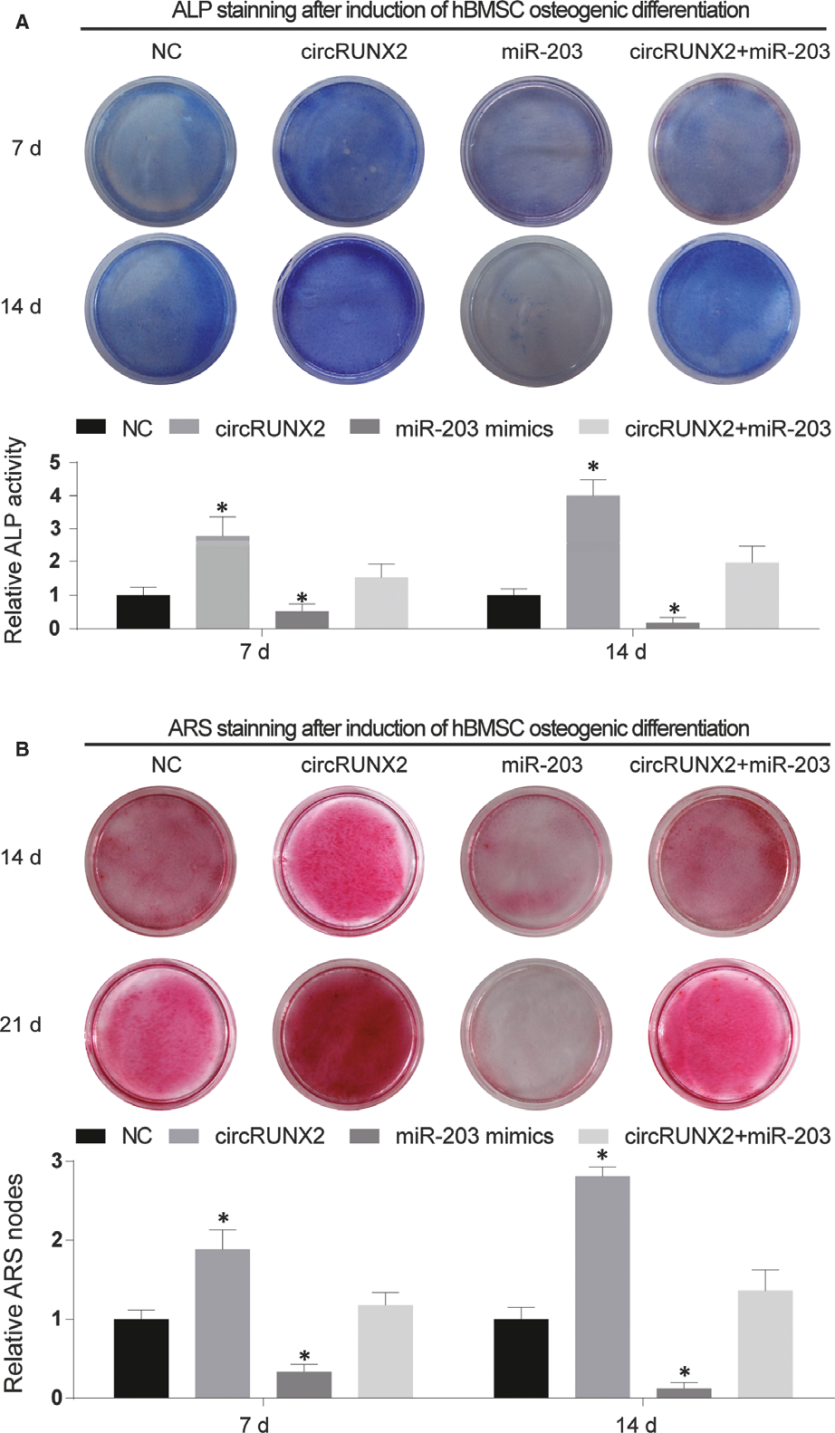
CircRUNX2 affected osteogenic differentiation through regulating miR‐203. (A and B) ALP staining and ARS staining were performed at 7th, 14th and 21th, respectively. *P <* *0.05

Furthermore, the differentiation level was measured by ALP and ARS staining assays in these four kinds of hBMSCs (Figure 6). After several days (7 days, 14 days and 21 days) of hBMSC osteogenic differentiation, ALP activity, and ARS nodes was dramatically higher in circRUNX3 group but significantly lower in miR‐203 group when compared with NC group. The rescued group showed a slight increase but no difference with NC group, which might imply that the target relationship existed between circRUNX2 and miR‐203.

## 4 DISCUSSION

RUNX2 is widely known as an important transcription factor in osteoblast differentiation. There are much more attentions towards the role of RUNX2 during the progress of osteogenic differentiation and other related regulatory genes, in order to figure out how osteogenic differentiation happens. For instance, the study of Shouhe et al revealed that Fra‐1 is a direct target of RUNX2, through combining to RUNX motif on promoter region of Fra‐1, during the early period of osteogenic differentiation of C2C12 myogenic progenitor cells.^13^ Yoon et al found that RUNX2 could be inhibited by NAA10 in the process of BMP‐2‐induced differentiation and bond growth.^18^ Some studies showed that RUNX2 expression is up‐regulated with RBM3 overexpression, which stimulated osteoblast differentiation; whereas the RUNX2 expression decreased in the presence of RBM3 inhibitor.^19^ However, the RUNX2‐related regulation mechanism is far from comprehensive. Herein, current study is aimed at investigating RUNX2‐related regulation system during the progress of osteogenic differentiation.

CircRNAs are identified as miRNA sponges to participate in the regulation of biological proccess, according to many researches. Tomas B et al revealed this funding firstly by reporting that there are over 70 conserved miRNA targeting sites on ciRS‐7. They demonstrated it with the fact that sex‐determining region Y (Sry) 9 functions as a miR‐138 sponge.^20^ Since then, a great deal of evidence confirmed above findings. Mechanical stress‐related circRNAs could regulate the tumour necrosis factor alpha expression level and influence chondrocyte ECM degradation process via targeting miR‐875.^16^ CircHIPK3 regulated cells growth by combining to miR‐124 and reducing its activity.^21^ Therefore, we continued to explore molecular mechanism during the progress of osteogenic differentiation with circRNA‐miRNA‐mRNA axis models.

In the past, some circRNAs, like circ19142 and circ5846, are reported to be involved in BMP2‐reduced osteoblast differentiation, through a circ19142/circ5846‐targeted miRNA‐mRNA axis.^6^ However, the role of circRNAs in this process, for instance, hsa‐circ‐0076694 (circRUNX2), remained to be researched. In this research, we focused on how circRUNX2 influenced osteoporosis and revealed the mechanism of osteogenic differentiation related to circRUNX2. We linked the correlation of circRUNX2 and RUNX2 and predicted their common targeting miRNA. What's more, we used, RNA pull down assay, RIP assay, RNA FISH assay, dual‐Luciferase Reporter assay, and functional experiments to verify our prediction. The present research revealed the molecular regulation mechanism of circRUNX2/miR‐203/RUNX2 axis during the progress of osteogenic differentiation, which will provide a novel therapy target for osteoporosis.

Although we tried to take all the factors into consideration in our experiments, some aspects could still be improved. For example, because of the difficulties in obtaining clinical samples, we only applied 10 osteoporosis specimens for our investigation. The conclusions would be more reliable on condition that there were more samples. Furthermore, there are several other bone differentiation related proteins detected in our experiments, like BSP, OCN, and OPN. Thus, next experiment might focus on how circRUNX2 regulated these proteins and enrich the mechanism of osteoblast differentiation.

## 5 CONCLUSION

This study mainly researches the effects of the circRUNX2 and its relations with other close genes. CircRUNX2 might prevent osteoporosis through regulating has‐miR‐203 and RUNX2. In order to get the more safe and effective therapies for osteoporosis, more circRNAs, mRNAs, and miRNAs should be researched to find out their effect and the links among them.

## CONFLICT OF INTEREST

The authors confirm that there are no conflicts of interest.
