# Supplementary figures and images for: CircRUNX2 through has‐miR‐203 regulates RUNX2 to prevent osteoporosis
Source: J Cell Mol Med. 2018 Oct 16;22(12):6112–21. doi: 10.1111/jcmm.13888 (PMC6237596; doi:10.1111/jcmm.13888)

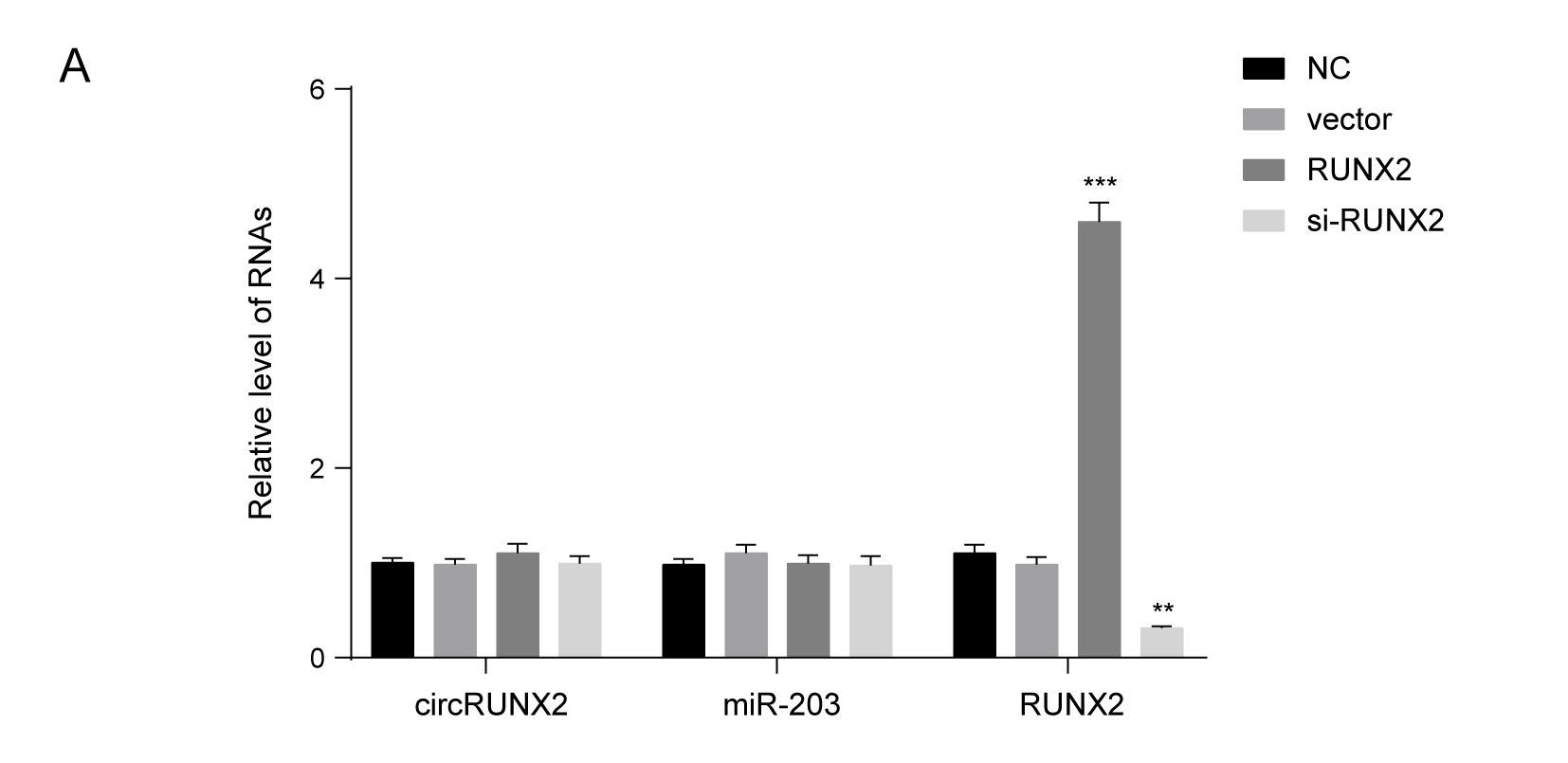

Supplement: Supplementary file 1 [file JCMM-22-6112-s001.tif]
